# Supplementary material for: Learning curve and surgical outcome of robotic assisted colorectal surgery with ERAS program
Source: Sci Rep. 2022 Nov 29;12:20566. doi: 10.1038/s41598-022-24665-w (PMC9709162; doi:10.1038/s41598-022-24665-w)
Supplement: Supplementary file 2 — Supplementary Information 2. [file 41598_2022_24665_MOESM2_ESM.zip › RAL Raw data-Ñ[▒K/113/HS_ñw╝╨░O▒Kñσ.pdf]

正式摘要(電子簽章：000000226198767)<br>W52 / W52 ☐ 外文標題(注意:無英文姓名-請補病人基本檔!)	☐【讀取】憑證	

臺中榮民總醫院

出院病歷摘要

轉入號： 轉出號：

[redacted]

(8)流水編號	
(9)入院日期	110/09/22 (2021) 直腸外科 W52 - 162
(10)轉科日期	
(11)出院日期	110/09/29 (2021) 住院天數計 7 日

診斷

- 入院 :1. S-colon cancer, cT3N1M0, stabe IIIB
  <Underlying disease>
  1. 3V-CAD s/p PCI at Tung's hospital on 2021/06/30
  2. Hypertension under medicine control
  3. Type II DM under medicine control (9/22HbA1C:5.3)
  4. Sleep apnea syndrome under CPAP use
- 出院 :1. S-colon cancer, cT3N1M0, stabe IIIB, s/p roboic-assisted anterior resection on 2021/09/23, pT3N1c
  <Underlying disease>
  1. 3V-CAD s/p PCI at Tung's hospital on 2021/06/30
  2. Hypertension under medicine control
  3. Type II DM under medicine control (9/22HbA1C:5.3)
  4. Sleep apnea syndrome under CPAP use

主訴

- bood-tinged stool for half year

病史

- This 63-year-old man has history of
  1. 3V-CAD s/p PCI at Tung's hospital on 2021/06/30
  2. Hypertension under medicine control
  3. Type II DM under medicine control (9/22HbA1C:5.3)
  4. Sleep apnea syndrome under CPAP use
  5. Anal fistula s/p fistulectomy in 1980s
  According to medical record and patient, he was suffered from bood-tinged stool for half year and colonoscopy survey(2021/06/11) revealed S-colon adenocarcinoma, moderately differentiated. Abdominal CT revealed compatible S-colon cancer, cT3N1M0. Operation was suggested. However, coronary artery calcificaiton was found and 2D ECHO also showed LV DISTAL 1/2 IVS HYPOKINESIA. Therefore he was discharged then and he underwent PCI therapy at Tung's hospital on 06/30. He had regular CV OPD follow up and was told hold Aspirin and Plavix use before surgery. As above problems, he was admitted for surgery.

體檢發現

-
  - General appearance :
    - easy looking , conscious clear , without cardiopulmonary distress
    - Vital signs自動帶入date 體溫(BT) 脈搏(P) 呼吸(R) 血壓(BP) 身高(BH) 體重(BW) BMI
      20210922 36.3℃ 76次/分 19次/分 125/74mmHg 168.8cm 77kg 27.0
    - Vital signs: B.P. 125/74 mmHg , P.R. 76 /min , R.R. 19 /min , B.T. 36.3 ℃
  - HEENT :
    - Eyes: no anemic conjunctiva, no icteric sclera, normal EOM
    - ENT: no otorrhea, no rhinorrhea, no erythematous mucosa, no injected tonsil, no deformity
  - Neck: supple, freely movable, trachea midline,no deformity, no engorged jugular vein , no palpable thyroid or lymph node enlargement
  - Chest:
    - Inspection: Grossly normal, symmetric and fully expansion, no accessory muscle used, no operation scar, no spider angiomata, no gynecomastia
    - Palpation: no palpable mass or nodule, no subcutaneous emphysema, no tenderness
    - Ausculation: clear breathing sound
  - Heart:
    - Percussion: normal heart size
    - Ausculation: regular heart beat, normal S1, S2, no gallop, no murmur
  - Abdomen:
    - Inspection: in
      - flat contour, symetrically, no scar, no stria, no dilated veins, no rashes
    - Ausculation: normoactive bowel sound, no abdominal bruits
    - palpation: soft, no muscle guarding or rigidity, no local tenderness, no rebounding pain, no palpable mass, no palpable pulsation
    - Percussion: no shifting dulllness, no hepatomegaly, no splenomagaly
  -

Peripheral pulsation :	CA	BA	RA	FA	PA	PTA	DPA
Right side :	++	++	++	++	++	++	++/+++
Left side :	++	++	++	++	++	++	++/+++

  - Genito-anus: no tenderness or palpable mass lesion or rectal shelf, normal external genitalia, normal anal tone, brown stool

CRS CONDITION

- Imaging studies(Sono , CT of abdomen and chest Films):
  - 檢驗項目:CT ABDOMEN-SURVEY 報告日期:1100615
    主 訴：
    Result:Y Finding: Imaging Report Form for Colorectal Carcinoma (AJCC 8th) 1. Imaging Date: 20210611 Imaging Modality, Imaging by CT scan Imaging Protocol: CT of whole abdomen post IV and oral contrast enhancement 2. Tumor location / Size Location: Sigmoid Size: Measurable: 5.5 cm (largest diameter) 3. Tumor invasion Tumor invades non-peritonealized pericolonic or perirectal tissues 4. Regional nodal metastasis N1 | N1b Two or three regional lymph nodes are positive. 5. Distant metastasis (In this study) No or Equivocal 6. Other findings: 1) Presence of segmental wall thickening with pericolonic fat stranding and small regional lymph nodes at S-colon, compatible with colon cancer. 2) Two surgical clips are noted at S-colon and D-colon. 3) No abnormal focal lesion in liver, GB, pancreas, bilateral kidneys and spleen. 4) Calcification of coronary arteries Impression: S-colon cancer
-
  - Lung

手術日期及方法

- 手術日期及方法20210923 Robot Anterior resection
- 手術結果及發現：
  - 1. A 4 x 5 cm, ulcerative tumor at S-colon. 2. No obvious ascites 3. Bilateral doughnuts : intact ; Air leak test: Done and no air leak was found

住院治療經過

- After admission, preoperative prepare based on ERAS protocol was done, he underwent robotic-assisted anterior resection on 2021/09/23. After surgery, he had regained conscious and tolerated well with PCA control. Post-op ERAS care was applied. He had semi-liquid oral diet smoothly and Foley removal at POD-2, turned off PCA control at POD-3, soft diet at POD-4. Penrose drainage tube was removed at POD-6. He was discharged.

合併症

- Nil

一般檢查

- 累積報告：

DATE	TIME	ALB	TP	BIL.T	BIL.D	ALKP	AST	ALT	LDH	AMY	LIPASE	NH3	Ferritin	UA
1100917	1203	4.1		0.4	0.1	67	15	10	132					
1100923	1554	2.6		0.90	0.29		13	9						
1100924	0527			0.9	0.3		18	15						
1100928	0852	3.4		0.45	0.13		25	10						

DATE	TIME	WBC	RBC	HGB	HCT	MCV	PLT	BLST	NEUT	BAND	SEG	LYM	MON	EOS	BAS
1100916	1021	5720		14.7			192		71.6			18.9	6.6	2.6	0.3
1100917	1203	6630	5.02	14.6	45.3	90.2	191		68.6			22.9	5.6	2.4	0.5
1100923	1555	7780	4.07	11.7	35.3	86.7	146		89.0			6.9	4.1	0.0	0.0
1100923	2313	7500	3.67	10.7	31.7	86.4	147		83.1			11.5	5.3	0.0	0.1
1100924	0519	5480	3.23	9.5	28.2	87.3	121		83.2			10.4	6.4	0.0	0.0
1100925	0901			9.2	27.3										
1100928	0908	5070	4.17	12.5	37.0	88.7	186		64.7			21.9	6.5	6.7	0.2

DATE	TIME	NA	K	CL	BUN	CREAT	eGFR	CA	Mg	P	CRP	ESR	PCT	D-Dimer	LACTATE
1100916	1022	141	4.4		22	0.91	89.44								22.5
1100917	1203	138	4.4		24	0.98		9.2							
1100923	1552														27.6
1100923	1554	135	3.9		16	0.87	94.2								
1100923	2313	140	3.9		16	0.92		7.7							
1100924	0522														7.1
1100924	0527	141	3.7		15	0.90	90.58								
1100925	0856	139	3.6					7.4							
1100928	0852	140	4.0		10	0.75	111.8	7.9							

DATE	TIME	GLUCOSE	HbA1C	CHOL	TG	HDL	LDL	KETONE	CK	CKMB	TRO-T	PT-P	APTT-P	NT-pro BNP
1100916	1022	102												
1100916	1056											11.4	30.1	
1100917	1203	111										10.9	30.3	
1100922	1710		5.3											
1100923	2313											11.5	29.9	

DATE	TIME	PH	PCO2	PO2	SO2	BEB	HCO3	TCO2	HGB	FIO2
1100923	1604	7.356	38.2	191.8	99.6	-4.1	20.9	22.1		

DATE	TIME	PH	BEB	SCVO2	PO2	HCO3	HGB	PCO2	TCO2
1100916	1021	7.404	1.5	94.9	74.7	26.5		43.4	27.9
1100924	0521	7.424	2.5	99.0	129.4	27.3		42.6	28.6

- 放射線報告 :檢驗項目 **:CHEST PA VIEW** 報告日期 **:1100927**

主　訴：
Result:N
Finding:
Suboptimal study due to inadequate inspiration
> The heart size is within normal limit
> Calcification of thoracic aorta
> Bilateral hilar prominence
> Increased insterstitial infiltration over bilateral lung fields
> Mild degenerative change of thoracic and lumbar spine with osteophytes formation.
> On central line
Impression:
As above description.

特殊檢查

病理報告

- 活體組織切片病理報告　手術日期：**110/09/23**　病理號：**S1030192**

診斷醫師:[illegible] 報告日期:110/09/30
Pathologic diagnosis:
1. Sigmoid colon, robotic anterior resection --- Adenocarcinoma, moderately differentiated. Tumor invaded to mesocolic soft tissue. The bilateral cut ends and mesocolic lymph nodes (0/19) are free of tumor.
2. IMA root, dissection --- Unremarkable lymph nodes (0/3).
3. Colon, cut end, distal, resection --- Unremarkable.
Ancillary study for diagnosis:
IHC stains for PMS2 and MSH6 are done on section H. Both markers are preserved.
Prognostic and predictive factor:
1. Tumor size: 5 cm.
2. Depth of invasion: Mesocolic soft tissue.
3. Histologic grade: G2: Moderately differentiated.
4. Angiolymphatic invasion: Not identified.
5. Perineural invasion: Not identified.
6. Tumor deposits (discontinuous extramural extension): Present, specify number of deposits: 2.
7. Macroscopic tumor perforation: Not identified.
8. Surgical Margins: a) Proximal Margin: Uninvolved by invasive carcinoma, distance of tumor from margin: 15.5 cm. b) Distal Margin: Uninvolved by invasive carcinoma, distance of tumor from margin: 1.5 cm. c) Radial or Mesenteric Margin: Uninvolved by invasive carcinoma, distance of tumor from margin: 3 cm.
9. Lymph node status: Uninvolved (0/22).
10. Extranodal involvement: Not identified.
11. Tumor regression grading S/P CCRT: No known presurgical therapy.
12. Type of polyp in which invasive carcinoma arose: Not identified.
13. Additional pathologic findings: Not identified.
14. Pathological TNM Stage: pT3N1c (According to the eighth edition, American Joint Committee on Cancer Staging Guidelines for Tumors).
15. TNM descriptors: Not applicable.

Gross description:
The specimen includes 1) a segment of colon, 22 cm in length, 6 cm in proximal cut end circumference, 5 cm in distal cut end circumference. There is a tan gray, centrally ulcerative and peripherally elevated tumor, 5x4 cm, noted 1.5 cm away from the distal cut end. The tumor is 3 cm to the radial margin grossly. The bilateral cut ends are free of tumor grossly. 2) “IMA root”, a piece of yellow soft tissue, 3x3x1 cm. 3) “distal cut end”, a piece of tan soft tissue, 3x2x2 cm. Representative part for section: A) specimen 2 B) specimen 3 C) proximal cut end D) radial margin E-J) tumor K-M) lymph nodes.
Note:
1. The interpretation of MMR panel and recommended studies are as below: (1) No loss of nuclear expression of MMR proteins: low probability of microsatellite instability-high (MSI-H) . (2) Loss of nuclear expression of MLH1 and PMS2: testing for methylation of the MLH1 promoter and/or mutation of BRAF is indicated (the presence of a BRAF V600E mutation and/or MLH1 methylation suggests that the tumor is sporadic and germline evaluation is probably not indicated; absence of both MLH1 methylation and of BRAF V600E mutation suggests the possibility of Lynch syndrome, and sequencing and/or large

- deletion/duplication testing of germline MLH1 may be indicated). (3) Loss of nuclear expression of MSH2 and MSH6: high probability of Lynch syndrome (sequencing and/or large deletion/duplication testing of germline MSH2 may be indicated, and, if negative, sequencing and/or large deletion/duplication testing of germline MSH6 may be indicated). (4) Loss of nuclear expression of MSH6 only: high probability of Lynch syndrome (sequencing and/or large deletion/duplication testing of germline MSH6 may be indicated). (5) Loss of nuclear expression of PMS2 only: high probability of Lynch syndrome (sequencing and/or large deletion/duplication testing of germline PMS2 may be indicated). (6) Please note: There are exceptions to the above IHC interpretations. These results should not be considered in isolation, and clinical correlation with genetic counseling is recommended to assess the need for germline testing.
  2. This case has been peer reviewed by two doctors.
  #T-59470_2 #M-81403_2 2 1105 S0000F
  #T-C4560_2 #M-09183_2 0 1105 000000

其它

出院時情況

- 3.改門診治療

出院指示

藥名	劑量	劑量單位	用法	頻次	數量	數量單位	現況	天數	單位
Plavix tab 75mg	1	TAB	PO	QD	7	TAB	TH	7	天
Mag. Oxide tab	2	TAB	PO	TID	42	TAB	TH	7	天
Pantoloc tab 40mg	1	TAB	PO	QDAC	7	TAB	TH	7	天
0.5mg Rivotril tab	1	TAB	PO	HS	7	TAB	TH	7	天
Ultracet tab	1	TAB	PO	Q6H	28	TAB	TH	7	天
Acertil tab 5mg	0.5	TAB	PO	QDAC	4	TAB	TH	7	天
TramTOR cap 50mg	1	CAP	PO	Q8HPRN	21	CAP	TH	7	天
1.25mg Bisoprolol tab	1	TAB	PO	QD	7	TAB	TH	7	天
Jardiance tab 10mg	0.5	TAB	PO	BID	7	TAB	TH	7	天
Mirtapine OD tab 30mg	1	TAB	PO	QN	7	TAB	TH	7	天
Urief tab 4 mg	1	TAB	PO	BID	14	TAB	TH	7	天

出院計劃

- 門診日期
  - date on 2021/10/06

主治醫師 2062F 蔣鋒帆 / 住院醫師 2336L 洪浚殿

正式摘要(電子簽章：000000226198767)

W52 / W52

doctorRank:B
